# Search for *NTRK1* proto-oncogene rearrangements in human thyroid tumours originated after therapeutic radiation

**DOI:** 10.1054/bjoc.1999.0920

**Published:** 2000-01-18

**Authors:** A Bounacer, M Schlumberger, R Wicker, J A Du-Villard, B Caillou, A Sarasin, H G Suárez

**Affiliations:** Laboratoire de Génétique Moléculaire UPR 42, Institut de Recherches sur le Cancer, C.N.R.S. IFC1, Villejuif Cedex, 94801, France; Institut Gustave Roussy, Villejuif Cedex, 94805, France

**Keywords:** thyroid, ionizing radiation, *NTRK1* proto-oncogene, rearrangements, *TRK* oncogene

## Abstract

Rearrangements of *NTRK1* proto-oncogene were detected in ‘spontaneous’ papillary thyroid carcinomas with a frequency varying from 5 to 25% in different studies. These rearrangements result in the formation of chimaeric genes composed of the tyrosine kinase domain of *NTRK1* fused to 5′ sequences of different genes. To investigate if the *NTRK1* gene plays a role in radiation-induced thyroid carcinogenesis, we looked for the presence of *NTRK1* -activating rearrangements in 32 human thyroid tumours (16 follicular adenomas, 14 papillary carcinomas and two lymph-node metastases of papillary thyroid carcinomas) from patients who had received external radiation, using the reverse transcription polymerase chain reaction, Southern blot and direct sequencing techniques. These data were compared with those obtained in a series of 28 ‘spontaneous’ benign and malignant thyroid tumours, collected from patients without a history of radiation exposure and four in vitro culture cell lines derived from ‘spontaneous’ thyroid cancers. Our results concerning the radiation-associated tumours showed that only rearrangements between *NTRK1* and *TPM3* genes (*TRK* oncogene) were detected in 2/14 papillary carcinomas and in one lymph-node metastasis of one of these papillary thyroid carcinomas. All the radiation-associated adenomas were negative. In the ‘spontaneous’ tumours, only one of the 14 papillary carcinomas and one of the four in vitro culture cell lines, derived from a papillary carcinoma, presented a *NTRK1* rearrangement also with the *TPM3* gene. Twenty-five of this series of radiation-associated tumours were previously studied for the *ras* and *RET/PTC* oncogenes. In conclusion, our data: (a) show that the overall frequency of *NTRK1* rearrangements is similar between radiation-associated (2/31: 6%) and ‘spontaneous’ epithelial thyroid tumours (2/32: 6%). The frequency, if we consider exclusively the papillary carcinomas, is in both cases 12%; (b) show that the *TRK* oncogene plays a role in the development of a minority of radiation-associated papillary thyroid carcinomas but not in adenomas; and (c) confirm that *RET/PTC* rearrangements are the major genetic alteration associated with ionizing radiation-induced thyroid tumorigenesis. © 2000 Cancer Research Campaign


*British Journal of Cancer* (2000) **82**(2), 308–314
© 2000 Cancer Research Campaign
Article no. bjoc.1999.0920

### Search for *NTRK1* proto-oncogene rearrangements in human thyroid tumours originated after therapeutic radiation

**A Bounacer[1], M Schlumberger[2], R Wicker[1], JA Du-Villard[1], B Caillou[2], A Sarasin[1] and HG Suárez[1]**

[1]Laboratoire de Génétique Moléculaire UPR 42, Institut de Recherches sur le Cancer, C.N.R.S. IFC1, 94801 Villejuif Cedex, France; [2]Institut Gustave Roussy, 94805 Villejuif Cedex, France

**Summary** Rearrangements of *NTRK1* proto-oncogene were detected in 'spontaneous' papillary thyroid carcinomas with a frequency varying from 5 to 25% in different studies. These rearrangements result in the formation of chimaeric genes composed of the tyrosine kinase domain of *NTRK1* fused to 5′ sequences of different genes. To investigate if the *NTRK1* gene plays a role in radiation-induced thyroid carcinogenesis, we looked for the presence of *NTRK1*-activating rearrangements in 32 human thyroid tumours (16 follicular adenomas, 14 papillary carcinomas and two lymph-node metastases of papillary thyroid carcinomas) from patients who had received external radiation, using the reverse transcription polymerase chain reaction, Southern blot and direct sequencing techniques. These data were compared with those obtained in a series of 28 'spontaneous' benign and malignant thyroid tumours, collected from patients without a history of radiation exposure and four in vitro culture cell lines derived from 'spontaneous' thyroid cancers. Our results concerning the radiation-associated tumours showed that only rearrangements between *NTRK1* and *TPM3* genes (*TRK* oncogene) were detected in 2/14 papillary carcinomas and in one lymph-node metastasis of one of these papillary thyroid carcinomas. All the radiation-associated adenomas were negative. In the 'spontaneous' tumours, only one of the 14 papillary carcinomas and one of the four in vitro culture cell lines, derived from a papillary carcinoma, presented a *NTRK1* rearrangement also with the *TPM3* gene. Twenty-five of this series of radiation-associated tumours were previously studied for the *ras* and *RET/PTC* oncogenes. In conclusion, our data: (a) show that the overall frequency of *NTRK1* rearrangements is similar between radiation-associated (2/31: 6%) and 'spontaneous' epithelial thyroid tumours (2/32: 6%). The frequency, if we consider exclusively the papillary carcinomas, is in both cases 12%; (b) show that the *TRK* oncogene plays a role in the development of a minority of radiation-associated papillary thyroid carcinomas but not in adenomas; and (c) confirm that *RET/PTC* rearrangements are the major genetic alteration associated with ionizing radiation-induced thyroid tumorigenesis. © 2000 Cancer Research Campaign

**Keywords:** thyroid; ionizing radiation; *NTRK1* proto-oncogene; rearrangements; *TRK* oncogene

The first study relating external beam radiation exposure during childhood and thyroid tumorigenesis was described in 1950 (Duffy and Fitzgerald, 1950). Since then, an increased incidence of thyroid cancers has been observed in several populations including atomic bomb survivors (Conrad et al, 1970), patients with a history of external radiation for benign or malignant conditions (Shore et al, 1985), and more recently in children from Belarus and Ukraine after the Chernobyl nuclear power plant explosion (Kazakov et al, 1992). However, little is known concerning the molecular mechanisms originating the radiation-associated thyroid tumours. Radiation is able to induce DNA strand breaks and deletions and stimulates aberrant recombination events, giving rise to chromosomal translocations and intra-chromosomal rearrangements (Roth et al, 1995). In 'spontaneous' papillary thyroid carcinomas, two proto-oncogenes, *RET* and *NTRK1*, which encode membrane tyrosine kinase receptors, were found activated by rearrangement with a variable frequency (Bongarzone et al, 1996). Recently, we and others have reported a high prevalence of *RET* rearrangements in thyroid tumours from patients who had received therapeutic or accidental radiation (Ito et al, 1994; Fugazzola et al, 1995; Klugbauer et al,1995; Bounacer et al, 1997; Nikiforov et al, 1997). These data suggested that radiation exposure may be, with a high frequency (more than 60%), a direct inducer of *RET* rearrangements. Studies concerning the research of alterations of other genes in radiation-associated thyroid tumours are also available for *ras, gsp* and *p53* (Wright et al, 1991; Challeton et al, 1995; Fogelfeld et al, 1996; Nikiforov et al, 1996). For NTRK1, there is only a recent study by Beimfohr et al (1999) concerning exclusively post-Chernobyl tumours.

The human *NTRK1* proto-oncogene (also called *TRKA*) is located on the q arm of chromosome 1 (Weier et al, 1995) and encodes one of the receptors of the nerve growth factor (NGF) (Kaplan et al, 1991; Klein et al, 1991). *NTRK1* gene transcripts have been detected exclusively in peripheral nervous ganglia, indicating that the gene plays a role in the nervous system development and function (Martin-Zanca et al, 1990). *NTRK1* was originally detected as an oncogene (named *TRK*) in a human colon carcinoma, following transfection of tumoural high molecular weight DNA in NIH 3T3 cells and focus formation (Martin-Zanca et al, 1986). This activated version of the proto-oncogene was generated by a somatic intrachromosomal rearrangement fusing the tyrosine kinase (TK) domain of *NTRK1* with 5′ sequences of the non-muscular tropomyosin gene (*TPM3*). *NTRK1* proto-onco-

*Received 5 March 1999*
*Revised 12 July 1999*
*Accepted 23 July 1999*

*Correspondence to:* HG Suárez gene rearrangements have been described also in human papillary thyroid carcinomas (PTC). In these tumours, the *NTRK1* oncogenic rearrangements are the consequence of the fusion of the TK domain of *NTRK1* with at least three different genes: the *TPM3* gene as found in the original *TRK* oncogene (Butti et al, 1995); the *TPR* (translocated promoter region) gene, also located on chromosome 1q and first identified as part of the *MET* oncogene (*TPR-MET*) (Park et al, 1986), giving rise at two different oncogenes: *TRK-T1* and *TRK-T2* (Greco et al, 1992); and the *TFG* (TRK-fused gene) gene which function is unknown and located on chromosome 3, originating the *TRK-T3* oncogene (Greco et al, 1995). All these oncogenic forms of *NTRK1* encode cytoplasmic chimaeric proteins which are constitutively phosphorylated on tyrosine. The frequency of *NTRK1* activation in 'spontaneous' thyroid tumours (exclusively in PTC) varies from 15 to 25% in tumours from Italian patients (Bongarzone et al, 1989; Greco et al, 1992; Butti et al, 1995) to less than 5% in French and Japanese studies (Wajjwalku et al, 1992; Said et al, 1994; Delvincourt et al, 1996).

To determine if the *NTRK1* proto-oncogene activating rearrangements play a role in thyroid radiation-induced carcinogenesis, we studied a series of benign and malignant human thyroid tumours which were obtained from patients who had received external radiation therapy. We compared these data with those obtained by us: (1) in a series of 'spontaneous' thyroid tumours, collected from patients without any history of radiation exposure and (2) with that previously obtained (Challeton et al, 1995; Bounacer et al, 1997) after the study of 25 of the radiation-associated tumours, looking for the presence of activated *ras* and *Ret* genes.

### MATERIALS AND METHODS

### Patients

Tumours were collected at the Gustave Roussy Institute (Villejuif, France) and were histologically classified according to the WHO recommendations (Hedinger et al, 1989). A total of 31 tumours obtained from patients with a history of external irradiation for benign or malignant conditions, were examined: 16 follicular adenomas, 14 PTC and two lymph-node metastases of PTC (LNMPTC) including one from a patient whose primary thyroid tumour was also studied (Table 1). The doses received by the thyroid have been calculated according to Diallo et al (1996), for 23/31 of our patients treated for their first benign or malignant condition in the Gustave Roussy Institute. As controls, we studied 28 'spontaneous' human thyroid tumours, collected from patients

**Table 1** *TRK* rearrangements in thyroid tumours from patients exposed to ionizing radiation

| Patient | Sex | Age at irradiation (yr) and dose (Gy)[a] | Cause | Age at tumour diagnosis (yr) | Histology[b] | *TRK* rearrangement research by RT-PCR |
|---|---|---|---|---|---|---|
| PE1[d e] | Female | 1/4.5 | Haemangioma | 20 | PTC | – |
| JE2[e] | Male | 26/14 | Hodgkin's disease | 36 | PTC | + |
| JE3[e] | Male | 26/14 | Hodgkin's disease | 36 | LNMPTC | + |
| AU4[e] | Female | 3/nd[c] | Bronchial cyst | 38 | PTC | – |
| MA5[e] | Female | 2/nd | Neck furuncle | 22 | PTC | – |
| GO6[e] | Male | 10/1.4 | Parotid tumour | 18 | PTC | – |
| VA7[e] | Female | 28/nd | Neck zona | 50 | PTC | – |
| DZ8[e] | Female | 7/2 | Neck tuberc. | 38 | PTC | – |
| SO9[e] | Female | 23/0.03 | Tonsil | 46 | PTC | – |
| TS10[e] | Male | 23/11 | Hodgkin's disease | 36 | PTC | – |
| AM11 | Male | 13/21 | Goitre | 57 | PTC | – |
| DE12 | Female | 32/16 | Breast carcinoma | 55 | PTC | – |
| PA13[e] | Female | 1/nd | Cutaneous angioma | 36 | LNMPTC | – |
| BO14 | Male | 26/nd | Zona upper limb | 33 | PTC | + |
| FO15[e] | Female | 5/0.001 | Benign rum | 33 | PTC | – |
| FA16 | Male | 8/23 | Goitre | 30 | PTC | – |
| PL17[d e] | Female | 12/nd | Acne | 42 | Macr. Ad. | – |
| SA18[e] | Male | 25/nd | Tonsil | 53 | Micr. Ad. | – |
| BO19[e] | Female | 10/12.5 | Hodgkin's disease | 30 | Mix. Ad. | – |
| PE20[e] | Female | 26/12 | Hodgkin's disease | 46 | Micr. Ad. | – |
| AK21 | Female | 23/11.5 | Lymphoma | 33 | Micr. Ad. | – |
| LA22[d] | Female | 4/15.5 | Hodgkin's disease | 18 | Mix. Ad. | – |
| RE23 | Female | 12/10.5 | Hodgkin's disease | 38 | Mix. Ad. | – |
| MA24[e] | Male | 13/13 | Hodgkin's disease | 34 | Micr. Ad. | – |
| AB25 | Female | 5/7.3 | Nephroblastoma | 25 | Micr. Ad. | – |
| OF26[e] | Male | 4/29.2 | Medulloblastoma | 21 | Micr. Ad. | – |
| CA27[e] | Female | 5/27.6 | Neuroblastoma | 29 | Macr. Ad. | – |
| JA28 | Male | 3/nd | Neck tuberc. | 39 | Micr. Ad. | – |
| RO29[e] | Male | 1/10 | Neuroblastoma | 27 | Micr. Ad. | – |
| PA30 | Male | 6/14 | Hodgkin's disease | 21 | Macr. Ad. | – |
| FA31 | Female | 27/10.5 | Hodgkin's disease | 34 | Mix. Ad. | – |
| AU32[e] | Female | 29/11.5 | Hodgkin's disease | 47 | Macr. Ad. | – |

[a]Dose received by the thyroid calculated according to Diallo et al. (1996). [b]PTC: papillary thyroid carcinoma; LNMPTC: lymph-node metastasis of papillary thyroid carcinoma; Macr. Ad.: macrofollicular adenoma.; Micr. Ad.: microfollicular adenoma; Mix. Ad.: mixed adenoma (samples from tumours with both macro- and microfollicular features). [c]nd: not done. [d]Samples positive for *ras* (Challeton et al, 1995). [e]Samples positive for *RET/PTC* (Bounacer et al, 1997 and this study).

*© 2000 Cancer Research Campaign*

without any history of radiation: 14 follicular adenomas and 14 PTC (Table 2). Four in vitro cultured human cell lines, three (K2, K5 and K8) derived from 'spontaneous' PTC and one (K7) from a follicular less-differentiated carcinoma (Challeton et al, 1997), were also screened.

The genetic material used in our study was extracted from frozen tissues of radiation-associated tumours (Table 1) in the case of patients MA5, PA13, PE20 and CA27; for all the other samples the tissues used were paraffin-embedded. Concerning 'spontaneous' tumours (Table 2), the genetic material was extracted in all the cases from frozen tissues, with the exception of CO1, CH4, FR7, SZ9, AL11, KR12, MA13, TA14, AM27 and FA28 for which the tissues were paraffin-embedded (Table 2).

### RNA extraction

RNA isolation from Duboss or Bouin fixed paraffin-embedded tissue samples, was performed according to a previously described procedure (Bounacer et al, 1997). Total RNA was extracted from frozen tissues, using the RNA-B™ technique (Bioprobe Systems, France) following the manufacturer's instructions. Total RNA was extracted from in vitro culture cells as described by Michelin et al (1993). The quality of the RNAs was controlled by reverse transcription polymerase chain reaction (RT-PCR) amplification using β-actin specific primers as described by Viglietto et al (1995).

### RT-PCR method for detecting TRK oncogenes

The reverse transcription reaction was performed as previously described (Bounacer et al, 1997) using half the volume of RNA extracted from paraffin-embedded tissue extracts or 1.5 μg of total RNA from fresh tissue extracts. One fourth of the cDNA was used for PCR amplification with outer primers. For the paraffin-embedded tissue extracts, a second round of PCR was done with nested primers using 1:10 of the first round PCR product. The PCR amplifications were performed as previously described (Bounacer et al, 1997), using an automatic thermocycler (GeneAmp, Perkin-Elmer, France). Ten μl of PCR product were electrophoresed in a 2% agarose gel. PCR primer sequences used in this study are given in Table 3.

**Table 2** *TRK* rearrangements in human 'spontaneous' thyroid tumours (**A**) and in vitro culture cell lines (**B**)

**A**

| Patient | Sex | Age at tumour diagnosis (years) | Histology[a] | *TRK* rearrangement research by RT-PCR | *TRK* rearrangement research by Southern blot |
|---|---|---|---|---|---|
| CO1[d] | Female | 37 | PTC | – | ND[b] |
| SE2[c d] | Female | 15 | PTC | – | – |
| QU3 | Male | 39 | PTC | – | – |
| CH4[d] | Female | 32 | PTC | ND | – |
| SE5 | Male | 75 | PTC | – | – |
| MA6 | Female | 46 | PTC | – | – |
| FR7 | Female | 27 | PTC | ND | – |
| BL8[d] | Female | 44 | PTC | – | – |
| SZ9 | Female | 39 | PTC | ND | + |
| UR10 | Male | 55 | PTC | – | – |
| AL11[c] | Male | 36 | PTC | ND | – |
| KR12 | Female | 13 | PTC | – | ND |
| MA13 | Male | 57 | PTC | ND | – |
| TA14 | Female | 30 | PTC | ND | – |
| DU15[c] | Female | 56 | Macr. Ad. | – | – |
| CO16[c] | Female | 22 | Macr. Ad. | – | – |
| RO17 | Female | 28 | Mix. Ad. | – | – |
| FA18 | Female | 43 | Micr. Ad. | – | – |
| CO19 | Female | 50 | Macr. Ad. | – | – |
| GH20[c] | Female | 37 | Macr. Ad. | – | – |
| ME21 | Female | 42 | Macr. Ad. | – | – |
| DE22 | Female | 29 | Micr. Ad. | – | – |
| GU23[c] | Female | 46 | Mix. Ad. | – | – |
| RE24 | Male | 69 | Mix. Ad. | – | – |
| SA25 | Female | 64 | Macr. Ad. | – | – |
| TH26[c] | Female | 37 | Mix. Ad. | – | – |
| AM27 | Female | 35 | Macr. Ad. | ND | – |
| FA28 | Female | 43 | Mix. Ad. | ND | – |

**B**

| Cell lines | Derived from | *TRK* rearrangement research by RT-PCR |
|---|---|---|
| K2 | PTC | – |
| K5 | PTC | + |
| K7 | FLDC[e] | – |
| K8 | PTC | – |

[a] Abreviations are the same as in Table 1. [b] ND: not done. [c] Samples positive for *ras* (Said et al, 1994). [d] Samples positive for *RET/PTC* (Bounacer et al, 1997). [e] Follicular less-differentiated carcinoma.

*© 2000 Cancer Research Campaign*

**Table 3** Sequences of the primers used in our experiments for RT-PCR

| | Primer sequences | Nucleotide positions[a] |
|---|---|---|
| *NTRK1 primers:* | | |
| TRK-F (forward) | 5′-TCAACAACGGCAACTACACG-3′ | 1145–1164 |
| TRK-R2 (outer reverse) | 5′-CTTGATGTGGTGAACACAGG-3′ | 1632–1651 |
| TRK-IR (in-nested reverse) | 5′-AACTTGTTTCTCCGTCCAC-3′ | 1565–1584 |
| *TPM3 primers:* | | |
| TPM-F2 (outer forward) | 5′-TGAGCAGATTAGACTGATGG-3′ | 474–493 |
| TPM-is (in-nested forward) | 5′-TGATAAACTCAAGGAGGCAG-3′ | 579–598 |
| *TPR primers:* | | |
| TPR-F2 (outer forward) | 5′-AGAACCAATGAGAGACTATC-3′ | 399–418 |
| TPR-is (in-nested forward) | 5′-GGATGAACTTCAAGCTTCTG-3′ | 512–531 |
| *TFG primers:* | | |
| TFG-es2 (outer forward) | 5′-TGATACTGTGGATGGTAGGG-3′ | 431–450 |
| TFG-F2 (in-nested forward) | 5′-AACAGTCTACTCAGGTTAT-3′ | 482–500 |

[a]Nucleotide positions of the primers are based on the published sequences of *NTRK1* (Martin-Zanca et al, 1989), *TRK* (Martin-Zanca et al, 1986), *TRK-T1* (Greco et al, 1992) and *TRK-T3* cDNA (Greco et al, 1995).

### DNA extraction and Southern blot analysis

Genomic DNA was extracted from the frozen tissues as described by Suárez et al (1990, 1991). Southern blot analysis was performed as previously described (Delvincourt et al, 1996) using as probes, a 1.2 kb *Bal*I-*Eco*RI fragment of pDM10-1 plasmid and a 2.7 kb *Kpn*I insert of pDM8 plasmid, specific respectively, for the tyrosine kinase domain of the *NTRK1* proto-oncogene and the tropomyosin sequences (Martin-Zanca et al, 1986). Both plasmids were kindly provided by Dr Martin-Zanca (Universidad de Salamanca, Spain).

### DNA sequence analysis

Direct sequencing of the amplified DNA fragments was carried out by the dideoxy-nucleotide method (Sanger et al, 1977) with $^{33}$P ATP, using the double strand DNA cycle sequencing system kit (Gibco-BRL, Life Technologies, France) and the same primers as those used in the amplification, following the manufacturer's conditions.

### RESULTS

### Presence of trk rearrangements in radiation-associated and 'spontaneous' human thyroid tumours

A total of 31 radiation-associated thyroid tumours (16 follicular adenomas, 14 PTC and two LNMPTC) (Table 1), were screened for the presence of *TRK*, *TRK-T1*, *TRK-T2* and *TRK-T3* chimaeric transcripts, using RT-PCR. As shown in Table 1, the majority of our patients (20/31) were irradiated at a young age (less than 14 years old) and the dose received at the thyroid gland varied from less than 1 to 29 Gy. *TRK* (*TPM3-NTRK1*) chimaeric transcripts were only detected in 2/14 PTC. In patient JE, the *TRK* rearrangement has been detected in both the primary tumour (JE2) and a lymph nodal metastasis (JE3) (Table 1). All the radiation-associated follicular adenomas were negative. Figure 1A illustrates results of positive tumours. The quality of the extracted RNAs is shown in Figure 1B. The *TRK* bands (254 bp) were observed only after a second round of PCR, probably reflecting the fact that these RNAs were prepared from a small volume of Bouin or Duboss fixed sample. The three positive cases studied by RT-PCR (including the LNMPTC of patient JE2) were confirmed by sequencing the cDNA and one example of these sequences is shown in Figure 2.

As controls, we studied 28 'spontaneous' human thyroid tumours, collected from patients without any history of radiation:

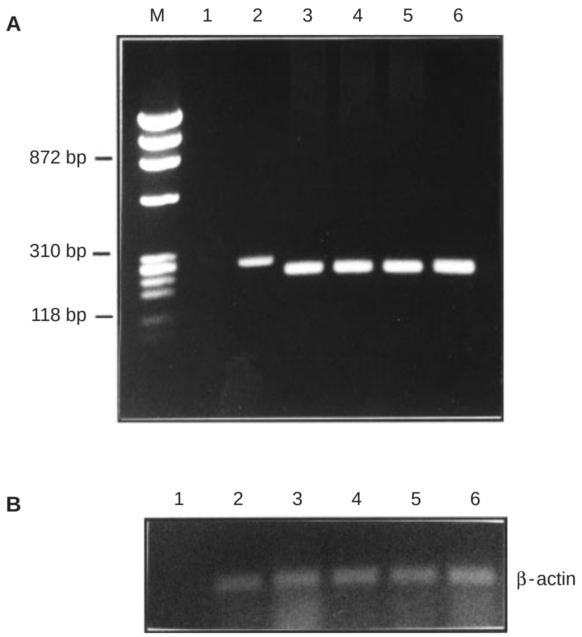

**Figure 1** Detection by RT-PCR of *NTRK1* rearrangements in the RNA of radiation-associated and 'spontaneous' human thyroid tumours. (**A**) Ethidium bromide-stained 2% agarose gel of second PCR round products. Lane 2: *NTRK1* expression in a 'spontaneous' tumour presenting a C-cell hyperplasia (positive control); lanes 3 and 4: *TRK* oncogene in the radiation-associated PTC of patient JE and its lymph-node metastasis respectively; lane 5: *TRK* oncogene in the radiation-associated PTC from patient BO14; lane 6: *TRK* oncogene in the cell line K5 derived from a human 'spontaneous' PTC. The predicted sizes of second PCR round fragments for *NTRK1* proto-oncogene and *TRK* oncogene are 265 bp and 254 bp respectively. Lane 1 shows the PCR amplification of RNA from a positive sample, which was not reverse transcribed prior to PCR amplification (negative control). M: Molecular weight marker ØX174/HaeIII. (**B**) The same RNAs in (**A**) were subjected to RT-PCR amplification using β-actin specific primers which generate an 82 bp PCR product. The products of the amplification were run on a 2% agarose gel and ethidium bromide staining of the gel is presented

© 2000 Cancer Research Campaign

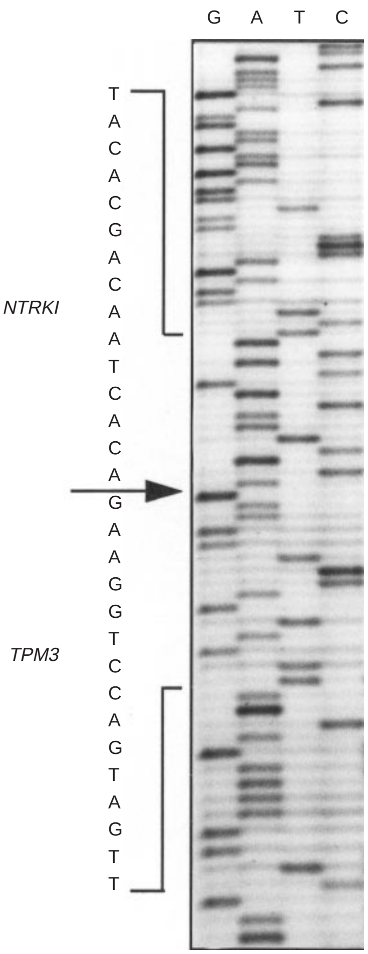

**Figure 2** Sequence analysis of RT-PCR cDNA prepared from *TRK*-positive radiation-associated tumour from patient JE2. The fusion point of the *TPM3* and *NTRK1* genes is indicated by an arrow

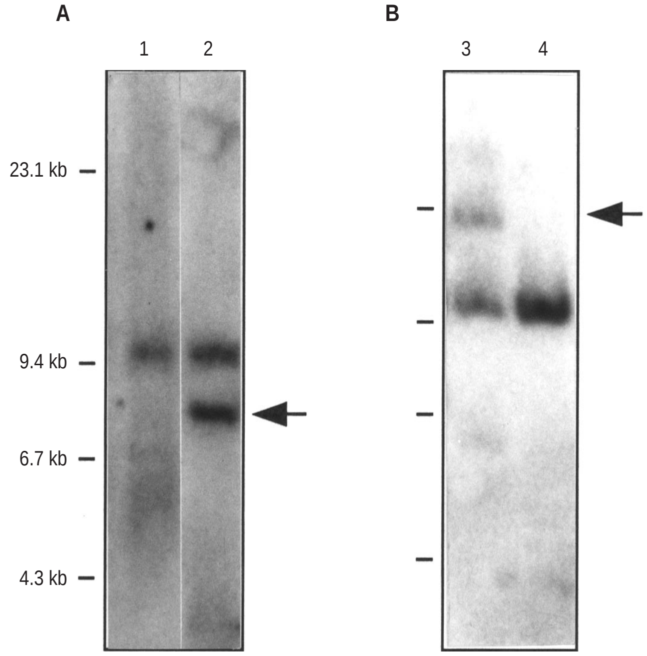

**Figure 3** Southern blot analysis of the *NTRK1* gene in: normal thyroid tissue (lanes 1 and 4) and 'spontaneous' PTC from patient SZ9 (lanes 2 and 3). The DNAs (20 μg) were digested with (**A**) *Bam*HI and hybridized with a probe derived from the tyrosine kinase domain of *NTRK1* (1.2 Kb *Bal* I-*Eco*RI fragment of pDM10-1 plasmid) and (**B**) with *Eco*RI and hybridized with a tropomyosin specific probe (2.7 Kb *Kpn*I insert of pDM8 plasmid). Co-electrophoresed λ*Hin*dIII DNA fragments serve as size markers. The arrows indicate the DNA fragments which defined the genetic rearrangement generating the *TRK* oncogene

14 follicular adenomas and 14 PTC (Table 2). Four in vitro cultured human cell lines, three (K2, K5 and K8) derived from 'spontaneous' PTC and one (K7) from a follicular less-differentiated carcinoma, were also screened. The presence of oncogenic *TRK*, *TRK-T1*, *TRK-T2* or *TRK-T3* rearrangements was investigated using the Southern blot and/or RT-PCR techniques (Table 2). Only one papillary carcinoma (patient SZ9) was found positive for *TRK* oncogene by Southern blot, using as probes a 1.2 kb *Bal*I-*Eco*RI fragment of pDM10-1 plasmid and a 2.7 kb *Kpn*I insert of pDM8 plasmid, specific respectively, for the tyrosine kinase domain of the *NTRK1* proto-oncogene and the tropomyosin sequences (Martin-Zancaet al, 1986) (Figure 3 A, B). All the 'spontaneous' follicular adenomas were negative. One of the four in vitro culture cell lines (K5) also presented a *TRK* rearrangement (*TPM3-NTRK1*), as shown by RT-PCR (Figure 1A) and cDNA sequence (data not shown).

Our results show that the overall frequency of *NTRK1* rearrangements is similar in radiation-associated and 'spontaneous' thyroid tumours (6%). When the PTC are considered separately, the frequencies are respectively 14% (2/14) and 12% (2/17) in the radiation-associated and 'spontaneous' tumours.

### Combined study of the *ras, RET/PTC* and *TRK* oncogenes in radiation-associated tumours

Twenty-five of the radiation-associated tumours have been also previously screened for the presence of *ras* mutations and *RET/PTC* rearrangements (Challeton et al, 1995; Bounacer et al, 1997) (Table 1). Three samples were positive for *ras*, 15 for *RET/PTC* and 2 for *TRK* (Table 1). The overall frequencies of *ras*, *RET* and *NTRK1* alterations in these radiation associated tumours are 12%, 60% and 8% respectively. Two of the radiation-associated tumours (patients PE1 and PL17) presented simultaneously a Ha-*ras* mutation and a *RET/PTC1* rearrangement (Table 1). In the remaining seven tumours which were not screened for *ras* (patient JE and patients numbered from 11 to 16), we looked by RT-PCR, for the presence of *RET/PTC* rearrangements. Three of these tumours [one PTC (FO15) and two LNMPTC (JE3 and PA13)] presented a *RET/PTC1* rearrangement (data not shown). Interestingly, one of them (JE2), presented simultaneously a *RET/PTC1* and a *TRK* rearrangement in both the primary tumour and its lymph-nodal metastasis (Table 1).

### DISCUSSION

In order to determine whether the *NTRK1* gene plays a role in radiation-associated thyroid tumorigenesis, we have studied 30 malignant and benign thyroid tumours and two lymph-node metastases, collected at the Gustave Roussy Institute (Villejuif, France) from patients with a history of external radiation (predominantly in childhood) for benign or malignant conditions. The results

 © 2000 Cancer Research Campaign obtained with these samples were compared: (1) with data obtained by screening 32 malignant and benign ‘spontaneous’ thyroid tumours (including four in vitro culture cell lines) and (2) with data previously obtained (Challeton et al, 1995; Bounacer et al, 1997) after the study of 25 of the radiation-associated tumours, looking for the presence of activated *ras* and *Ret* genes.

In our study, carried out using the RT-PCR and Southern blot techniques, a similar frequency of *NTRK1/TPM3* rearrangements was observed in radiation-associated and ‘spontaneous’ samples (6%). These data are similar to that recently published by Beimfohr et al (1999) studying a series of 81 tumours of children from Belarus who had been exposed to radiactive iodine after the Chernobyl reactor accident. The *NTRK1*-activating rearrangement was also observed in a lymph-node metastasis of one of the radiation-associated PTC (sample JE3: patient JE). In contrast to *RET*, relatively few studies have been devoted to *NTRK1* activation in ‘spontaneous’ thyroid tumours (Bongarzone et al, 1989; Greco et al, 1992, 1995; Wajjwalku et al, 1992; Said et al, 1994; Butti et al, 1995; Delvincourt et al, 1996). In this type of tumour, it has been shown that *NTRK1* rearrangements seem to be present exclusively in PTC with a frequency varying from 25% in Italian studies (Bongarzone et al, 1989; Greco et al, 1992; Butti et al, 1995) to less than 5% in French and Japanese studies (Wajjwalku et al, 1992; Said et al, 1994; Delvincourt et al, 1996). This difference may be the consequence of geographical factors, as suggested by Delvincourt et al (1996) studying a homogeneous population from the Champagne-Ardennes region of France. However, the possibility cannot be excluded that using the technique of transfection of high molecular weight tumour DNA in 3T3 cells, as was the case in the Italian studies, Delvincourt et al (1996) and Said et al (1994) would have also found a higher frequency of activation and perhaps new *NTRK1* chimaeric genes.

Up to date, there is no report concerning *NTRK1* activation in radiation-associated thyroid tumours. In our present study, the overall frequency of *NTRK1* rearrangements considering only the radiation-associated and ‘spontaneous’ PTC, is 14% (2/14) and 12% (2/17) respectively. In contrast with results previously reported by us concerning the *RET/PTC* oncogene (Bounacer et al, 1997), all the radiation-associated follicular adenomas were negative for the presence of *NTRK1* activating rearrangements, in agreement with previous and present data concerning ‘spontaneous’ thyroid follicular adenomas (Wajjwalku et al, 1992; Said et al, 1994; Delvincourt et al, 1996).

The type of activating rearrangement of the *NTRK1* proto-oncogene observed by us in our radiation-associated and ‘spontaneous’ thyroid tumours, involved exclusively the *TPM3* gene. Little is known about the mechanism by which the *NTRK1* proto-oncogene is damaged by genotoxic agents, to generate a chimaeric gene. Until present, the only study carried out to characterize the sequence of the genomic regions involved in the *NTRK1* activating rearrangements, has been done by Butti et al (1995) in three ‘spontaneous’ PTC and concerns exclusively the *TRK* oncogene (*TPM3-NTRK1*). These authors showed that the different breakpoints occurred in intronic regions of both genes. They identified in these regions the presence of some recombinogenic elements including palindromes, direct and inverted repeats and Alu sequences. However, the significance of these results in the process of rearrangement after irradiation is still unknown, because there are no similar studies concerning thyroid radiation-associated tumours. Whether or not the mechanism of rearrangement is the same in ‘spontaneous’ and radiation-associated tumours, will probably be elucidated studying the breakpoints in both types of tumours.

Twenty-five of our radiation-associated tumours were previously screened for the presence of *ras* mutations and *RET/PTC* rearrangements (Challeton et al, 1995; Bounacer et al, 1997) (Table 1). The overall frequencies of *ras*, *RET* and *NTRK1* alterations in these radiation associated tumours are 12%, 60% and 8% respectively. This result confirms, as previously reported by us for tumours originated after therapeutic radiation (Bounacer et al, 1997) and by others in tumours appearing after the Chernobyl fallout (Ito et al, 1994; Fugazzola et al, 1995; Klugbauer et al, 1995; Nikiforov et al, 1997), that *RET* oncogenic activation by rearrangement represents the major genetic lesion associated with radiation-induced thyroid tumorigenesis. Three of the radiation-associated tumours presented simultaneously two different genetic alterations: in two cases a *RET/PTC1* rearrangement and a Ha-*ras* mutation (patients PE1 and PL17) and in one case a *RET/PTC1* and a *TRK* rearrangement (patient JE2) (Table 1). It is tempting to speculate about an eventual mechanism of cooperation between the simultaneously altered *ras* and *RET* genes in the initiation or progression of our human radiation-associated tumours, as previously described by Santoro et al (1993) studying an in vitro culture rat thyroid epithelial cell line. The fact that only a low number of our positive radiation-associated tumours present two simultaneous genetic alterations (3/25), pleads in favour of an alternative role of the *ras*, *RET* and *NTRK1* genes in the induction of the tumorigenic process. However, an eventual cooperation between these genes and/or with other unknown genes must not be neglected.

In conclusion, taken together our data: (a) show that the *NTRK1* proto-oncogene is activated by rearrangement with a similar frequency in ‘spontaneous’ and radiation-associated thyroid tumours; (b) show that the *NTRK1* proto-oncogene activating rearrangements play a role in the development of a minority of radiation-associated PTC but not in adenomas and (c) confirm that *RET* oncogenic activation by rearrangement is the major genetic event associated with ionizing radiation-induced thyroid tumorigenesis.

### ACKNOWLEDGEMENTS

We are indebted to M Chaker for the typewriting of the manuscript and L Daya-Grojean for the critical reading of the English form. This work was supported by grants from CNRS (Centre National de la Recherche Scientifique sur le Cancer, France), ARC (Association de la Recherche sur le Cancer, France), Ligue Nationale Française contre le Cancer, Fondation de France, Ministère de l’Education et de la Recherche (to HG Suárez) and Project JSP4CT940090 of the European Community (to M Schlumberger). A Bounacer held a fellowship from ARC.

### REFERENCES

Beimfohr C, Klugbauer S, Demidchick E, Lengfelder E and Raber HM (1999) NTRK1 re-arrangement in papillary thyroid carcinomas of children after the Chernobyl reactor accident. *Int J Cancer* **80**: 842–847

Bongarzone I, Pierotti M, Monzini N, Mondellini P, Manenti G, Donghi R, Pilotti S, Grieco M, Santoro M, Fusco A, Vecchio G and Della-Porta G (1989) High frequency of activation of tyrosine kinase oncogenes in human papillary thyroid carcinoma. *Oncogene* **4**: 1457–1462

Bongarzone I, Fugazzola L, Vigneri P, Mariani L, Mondellini P, Pacini F, Basolo F, Pinchera A, Pilotti S and Pierotti MA (1996) Age-related activation of the tyrosine kinase receptor protooncogenes RET and NTRK1 in papillary thyroid carcinoma. *J Clin Endocrinol Metab* **81**: 2006–2009

*© 2000 Cancer Research Campaign*

Bounacer A, Wicker R, Caillou B, Cailleux AF, Sarasin A, Schlumberger M and Suárez HG (1997) High prevalence of activating ret proto-oncogene rearrangements, in thyroid tumors from patients who had received external radiation. *Oncogene* **15**: 1263–1273

Butti MG, Bongarzone I, Ferraresi G, Mondellini P, Borrello MG and Pierotti MA (1995) A sequence analysis of the genomic regions involved in the rearrangements between TPM3 and NTRK1 genes producing TRK oncogenes in papillary thyroid carcinomas. *Genomics* **28**: 15–24

Challeton C, Bounacer A, Du Villard JA, Caillou B, De Vathaire F, Monier R, Schlumberger M and Suárez HG (1995) Pattern of ras and gsp oncogene mutations in radiation-associated human thyroid tumors. *Oncogene* **11**: 601–603

Challeton C, Branea F, Schlumberger M, Gaillard N, de Vathaire F, Badie C, Antonini P and Parmentier C (1997) Characterization and radiosensitivity at high or low dose rate of four cell lines derived from human thyroid tumors. *Int J Radiat Oncol Biol Phys* **37**: 163–169

Conrad RA, Dobyns BM and Sutow WW (1970) Thyroid-neoplasia as late effect of exposure to radioactive iodine in fallout. *JAMA* **214**: 316–324

Delvincourt C, Patey M, Flament JB, Suarez HG, Larbre H, Jardillier JC and Delisle MJ (1996) Ret and trk proto-oncogene activation in thyroid papillary carcinomas in French patients from the Champagene-Ardenne region. *Clin Biochem* **29**: 267–271

Diallo I, Lamon A, Shamsaldin A, Grimaud E, De Vathaire F and Chavaudra J (1996) Estimation of the radiation dose delivered to any point outside the target volume per patient treated with external beam radiotherapy. *Radiother Oncol* **38**: 269–271

Duffy BJJ and Fitzgerald PJ (1950) Cancer of the thyroid in children: a report of 28 cases. *J Clin Endocrinol Metab* **10**: 1296–1308

Fogelfeld L, Bauer TK, Schneider AB, Swartz JE and Zitman R (1996) p53 gene mutations in radiation-induced thyroid cancer. *J Clin Endocrinol Metab* **81**: 3039–3044

Fugazzola L, Pilotti S, Pinchera A, Vorontsova TV, Mondellini P, Bongarzone I, Greco A, Astakhova L, Butti MG, Demidchik EP, Pacini F and Pierotti MA (1995) Oncogenic rearrangements of the RET proto-oncogene in papillary thyroid carcinomas from children exposed to the Chernobyl nuclear accident. *Cancer Res* **55**: 5617–5620

Greco A, Pierotti MA, Bongarzone I, Pagliardini I, Lanzi C and Della-Porta G (1992) Trk-T1 is a novel oncogene formed by the fusion of tpr and trk genes in human papillary thyroid carcinomas. *Oncogene* **7**: 237–242

Greco A, Mariani C, Miranda C, Lupas A, Pagliardini S, Pomati M and Pierotti MA (1995) The DNA rearrangement that generates the TRK T3 oncogene involves a novel gene on chromosome 3 whose product has a potential coiled-coil domain. *Mol Cell Biol* **15**: 6118–6127

Hedinger C, Williams ED and Sobin LH (1989) The WHO histologic classification of thyroid tumours: a commentary on the second edition. *Cancer* **63**: 908–911

Ito T, Seyama T, Iwamoto KS, Mizuno T, Tronko ND, Komissarenko IV, Cherstovoy ED, Satow Y, Takeichi N, Dohi K and Akiyama M (1994) Activated Ret oncogene in thyroid cancers of children from areas contamined by Chernobyl accident. *Lancet* **344**: 259

Kaplan DR, Hempstead BL, Martin-Zanca D, Chao MV and Parada LF (1991) The trk proto-oncogene product a signal transducing receptor for nerve growth factor. *Science* **252**: 554–558

Kazakov VK, Demidchik EP and Astakhova LN (1992) Thyroid cancer after Chernobyl. *Nature* **395**: 21

Klein R, Jing S, Nanduri V, O'Rourke E and Barbacid M (1991) The trk proto-oncogene encodes a receptor for nerve growth factor. *Cell* **65:** 189–197

Klugbauer S, Lengfelder E, Demidchik EP and Rabes HM (1995) High prevalence of RET rearrangement in thyroid tumors of children from Belarus after the Chernobyl reactor accident. *Oncogene* **11**: 2459–2467

Martin-Zanca D, Hughes SH and Barbacid M (1986) A human oncogene formed by the fusion of truncated tropomyosin and protein tyrosine kinase sequences. *Nature* **319**: 743–748

Martin-Zanca D, Oskam R, Mitra G, Copeland T and Barbacid M (1989) Molecular and biochemical characterization of the human TRK proto-oncogene. *Mol Cell Biol* **9**: 24–33

Martin-Zanca D, Barbacid M and Parada L (1990) Expression of the trk protooncogene is restricted to the sensory cranial and spinal ganglia of neural crest origin in mouse development. *Genes Dev* **4**: 683–694

Michelin S, Daya-Grosjean L, Sureau F, Saïd S, Sarasin A and Suárez HG (1993) Characterization of c-met proto-oncogene actived in human xeroderma pigmentosum cells after treatment with N-methyl-N′-nitro-N-nitrosoguanidine (MNNG). *Oncogene* **8**: 1983–1991

Nikiforov YE, Nikiforova MN, Gnepp DR and Fagin JA (1996) Prevalence of mutations of ras and p53 in benign and malignant thyroid tumours from children exposed to radiation after Chernobyl nuclear accident. *Oncogene* **13**: 687–693

Nikiforov YE, Rowland JM, Bove KE, Monforte-Muñoz H and Fagin J (1997) Distinct pattern of ret oncogene rearrangements in morphological variants of radiation-induced and sporadic thyroid papillary carcinomas in children. *Cancer Res* **57**: 1690–1694

Park M, Dean M, Cooper CS, Schmidt M, O'Brien SJ, Blair DG and Van de Woude GF (1986) Mechanisms of met oncogene activation. *Cell* **45**: 895–904

Roth D, Lindahl T and Gellert M (1995) Repair and recombination. How to make ends meet. *Curr Biol* **5**: 496–499

Said S, Schlumberger M and Suárez HG (1994) Oncogenes and anti-oncogenes in human epithelial thyroid tumours. *J Endocrinol Invest* **17**: 371–379

Sanger F, Nicklen S and Coulson AR (1977) DNA sequencing with chain terminating inhibition. *Proc Natl Acad Sci USA* **82**: 488–492

Santoro M, Melillo RM, Grieco M, Berlingieri MT, Vecchio G and Fusco A (1993) The *TRK* and *RET* tyrosine kinase oncogenes cooperate with *ras* in the neoplastic transformation of a rat thyroid epithelial cell line. *Cell Growth Differ* **4**: 77–84

Shore RE, Woodard E, Hildreth N, Dvoresky P, Hempelmann L and Pasternack B (1985) Thyroid tumours following thymus irradiation. *J Natl Cancer Inst* **74**: 1177–1184

Suárez HG, Du Villard JA, Severino M, Caillou B, Schlumberger M, Tubiana M, Parmentier C and Monier R (1990) Presence of mutations in all three ras genes in thyroid tumors. *Oncogene* **5**: 565–570

Suárez HG, Du Villard JA, Caillou B, Schlumberger M, Parmentier C and Monier R (1991) Gsp mutations in human thyroid tumours. *Oncogene* **6**: 677–679

Viglietto G, Chiappetta G, Martinez-Tello FJ, Fukunaga FH, Tallini G, Rigopoulou D, Visconti R, Mastro A, Santoro M and Fusco A (1995) RET/PTC oncogene activation is an early event in thyroid carcinogenesis. *Oncogene* **11**: 1207–1210

Wajjwalku W, Nakamura S, Hasegawa Y, Miyazaki K, Satoh Y, Funahashi H, Matsuyama M and Takahashi M (1992) Low frequency of rearrangements of the ret and trk proto-oncogenes in Japanese papillary carcinomas. *Jpn J Cancer Res* **83**: 671–675

Weier H-UG, Rhein AP, Shadravan F, Collins C and Polikoff D (1995) Rapid physical mapping of the human trk protooncogene (NTRK1) to human chromosome 1q21–q22 by P1 clone selection, fluorescence in situ hybridization (FISH), and computer-assisted microscopy. *Genomics* **26**: 390–393

Wright P, Williams ED, Lemoine NR and Wynford-Thomas D (1991) Radiation associated and 'spontaneous' human thyroid carcinomas show different pattern of ras oncogene mutation. *Oncogene* **6**: 471–473

*© 2000 Cancer Research Campaign*
